# Tolosa-Hunt syndrome: focus on MRI findings and diagnostic criteria

**DOI:** 10.1186/1129-2377-14-S1-P157

**Published:** 2013-02-21

**Authors:** T Özbenli, K Akpýnar, H Doðru, L Ýncesu

**Affiliations:** 1 Ondokuz Mayýs University, Turkey; 2 Ondokuz Mayýs University, UK

## Introduction

Tolosa-Hunt Syndrome(THS) is a rare disorder characterized by periorbital or hemicranial pain, ipsilateral oculumotor paralysis and prompt response to steroids [1,2].

## Purpose/background/objectives

The etiology of THS is still unknown [3]. According to the diagnostic criteria demonstration of the presence of granuloma through MR imaging and/or biopsy is mandatory for diagnosis. The initial standard MR imaging may not be sufficient for diagnosis.

## Methods

According to the criteria revised in 2004 by the International Headache Society, seven cases diagnosed as THS have been assessed from the perspectives of type, age, symptoms and findings, accompanying diseases, MRI techniques used, localization of the determined lesion, response to the treatment and clinical progress. Routine biochemical tests, cranial MRI and CT angiographies were done on each patient, and lumbar puncture were also carried out on four.

## Results

Four of seven cases diagnosed are males. Age average of patients is 45,7 + 18,1 (25-69). In three of seven cases, cranial nerve paralysis of the third, in one case the fourth, another in sixth, two with fifth, and one with seventh nerve, and in two cases the third, fourth and/or sixth cranial nerves were determined to be bunched. In all cases a response was obtained within 24-72 hours to the corticosteroid treatment. Four out of seven cases had recurrence.

## Conclusion

Dynamic contrast enhanced MRI may be necessary to demonstrate the presence of granuloma. THS is a diagnosis of exclusion requiring careful patient evaluation to rule out vascular causes, tumour, or other forms of inflammation in the region of the cavernous sinus. In some cases, even though MRI is normal, the most logical diagnosis is THS. Patients categorized in this group may be as diagnosed as "probable THS" and follow-up at least two years until the necessary MRI methods are clearly defined in the IHS diagnostic criteria.
